# Observations Relative to the History of the Operation of Lithotomy

**Published:** 1819-11

**Authors:** John Lawrence


FOR THE LONDON MEDICAL AND PHYSICAL JOURNAL.

### *Observations relative to the History of the Operation of Lithotomy.*

By JOHN LAWRENCE, Esq.

LOOKING over a late publication of Mr. Carpue, on the high operation in Lithotomy, I find he dates the commencement of that practice in England from the year 1719, several years previously to the experiments of Cheselden. Now, to the best of my recollection, there is an author who in this case challenges priority over every other Englishman;—an author, in whom it is no small difficulty to decide whether vulgarity, conceit, or real sterling talent, predominate. I allude to Henry Bracken, M.D. *our* celebrated, experienced, and really profound, writer on the *res veterinaria*, who, I think, in his *Lithiasis Anglicana*, a tract to be found in some old medical collection, which I have not seen these thirty years, makes the above pretension. He says, he was the first man in England who practised the high operation; that he did so with success, and in consequence recommends it. Now, I apprehend there is a probable ground for his pretension, as he attended the Hôtel-Dieu at Paris, and was consequently acquainted with the discovery of *Frere Jaques;* and, as this might well be about the year 1712 or 1713, the doctor might have made his essay in England immediately afterwards.

Having no pretensions to a knowledge of the distinctive merits of the subject, the above observations are tendered as mere matter of historical record.

*Bridgewater-street, Sommers Town; Sept.* 16, 1819.

